# Comparison of factor structures of the Pittsburgh sleep quality index between mid- and late pregnancy among Korean women: a cross-sectional study

**DOI:** 10.1186/s12884-022-05024-z

**Published:** 2022-09-08

**Authors:** Hyejung Lee, Ki-Eun Kim, Mi-Young Kim, Chang Gi Park

**Affiliations:** 1 Mo-Im Kim Nursing Research Institute, Yonsei University College of Nursing, Seoul, Korea; 2 College of Nursing, Yonsei University, Seoul, 03722 Korea; 3 College of Nursing, Woosuk University, Jeonju-si, Jeollabuk-do Korea; 4 College of Nursing, The University of Illinois at Chicago, Chicago, IL USA

**Keywords:** Sleep quality, Pregnancy, Depression, Factor analysis, Korea

## Abstract

**Background:**

Sleep disturbance in pregnant women needs to be accurately assessed in a timely manner during pregnancy, to receive assessment-driven accurate intervention. This study aims to compare the factor structure of the Pittsburgh Sleep Quality Index (PSQI) among women in mid- and late pregnancy and evaluate the psychometric features of the Korean version of the PSQI.

**Methods:**

The survey questionnaire with the PSQI, Center for Epidemiological Studies Depression scale, and pregnancy stress, was completed by 281 women in mid- or late pregnancy. Exploratory factor analysis determined the best factor structure of the PSQI, and the Pearson correlation coefficient examined a convergent validity with depressive symptoms and pregnancy stress. Internal consistency was examined using Cronbach’s alpha.

**Results:**

In both mid- and late-pregnancy women, a two-factor structure model was identified**.** However, each factor’s different components were named differently. For women in mid-pregnancy, it was named “quantitative sleep quality” and “subjective sleep quality,” and for those in later stages of pregnancy, they were named “perceived sleep quality” and “daily disturbance.” The PQSI score showed a significantly positive correlation between depressive symptoms and pregnancy stress in mid- (r = 0.57 and r = 0.39, respectively), and late pregnancy (r = 0.48 and 0.28, respectively). The overall Cronbach’s alpha for the PSQI was 0.63.

**Conclusions:**

The Korean version of the PSQI demonstrated excellent construct and convergent validity, making it suitable to assess the sleep quality of women in middle to late stages of their pregnancy. The PSQI was found to have a two-factor structure in the mid-and late pregnancy, but the components were different. As sleep quality changes with each gestational stage, factors affecting it during mid- and late pregnancy need to be separately examined. It will make it easier for medical professionals to provide pregnant women sleeping irregularly, with the right kind of intervention.

## Background

Sleep disturbance in pregnant women has been of concern due to its intensifying prevalence and potential negative obstetric and neonate outcomes [1, 2]. A recent meta-analysis reported that approximately 45.7% of pregnant women experienced disturbed sleep during their pregnancy [1]. Fragmented sleep, difficulty in initiating sleep, and frequent awakening are common complaints among pregnant women [2]. These may be due to pregnancy-related physical and hormonal changes [2, 3] — these are expected and are, generally, temporary [4]; however, some pregnant women experience detrimental sleep problems that may lead to adverse obstetric and neonatal outcomes [5–7]. Sleep disturbances in early pregnancy predicted depressive symptoms in late pregnancy [8] and postnatal depression [1, 9]. The relationship between poor sleep quality and depressive symptoms was significant only in pregnant women above 30 years of age [9]. Sleep disturbance in pregnant women can be unique in terms of its temporary nature and association with the progression of pregnancy. However, compared to other clinical and non-clinical populations, this issue has been less frequently examined in pregnant women.

Of the available sleep assessment scales, the Pittsburgh Sleep Quality Index (PSQI) is the scale that is most extensively used to evaluate sleep experience and quality among pregnant women [7, 10]. This self-report questionnaire originally developed to measure the sleep health in the general population [11], consists of seven components that assess specific features of sleep, such as subjective sleep quality, latency, duration, efficiency, disturbance, medication use for sleep, and daytime dysfunction. The PSQI has been extensively translated to various languages, including Spanish, French, Chinese, Japanese, and Korean, and its psychometric properties have been validated in the respective populations and countries [12]. The scale was originally developed as a single-factor scale, however, the number of factors revealed in the study has varied from one to four factors [10]. The Spanish version of the scale was verified with pregnant women in the early gestational period [13] and a three-factor structure model was confirmed to include factors of sleep quality, efficiency, and medication use. Qiu et al. [8] reported a two-factor structure among healthy women in early pregnancy where the component of subjective sleep quality was cross-loaded on both factors. Even though women in early pregnancy were examined in both studies, the factors revealed were different; this suggests that differences in culture, demographics, and linguistics (translational process) may affect the interpretation of the domain and global scores of the PSQI. Thus, the dimensionality of the PSQI representing the sleep quality of a target population requires examination. The dimension can then be applied to the respective population to understand the score’s practical meaning and develop appropriate guidelines and interventions in clinical and community settings.

Sleep disturbance is very common among pregnant Korean women, and it has been observed that sleep deterioration significantly worsens from the seventh to the ninth month [2, 14]. Therefore, this study is designed to compare the factor structures among women in different gestational stages such as mid- and late pregnancy in Korea.

## Methods

### Study participants and design

A cross-sectional study design was used. Participants were women recruited from The Maternity School, an education program offered in a tertiary hospital located in Seoul, Korea. The Maternity School was a one-day program designed to provide useful information regarding healthy pregnancy and preparation for childbirth. In addition, several booths were set up outside the auditorium to advertise the commercial products related to pregnancy. Inclusion criteria were that the participants had to be over 20 years of age, with no known current illnesses, and pregnant, as confirmed by a physician. No exclusion criteria were applied.

### Data collection and measures

During the break between the educational lectures, a research booth was opened, and the principal researcher and assistants explained the study purpose and procedure to potential participants who showed interest in the study. Thereafter, the researchers obtained written informed consent from those who decided to participate in the study. Participants were asked to complete the survey questionnaire on the spot; in case they were unable to complete it then, they were asked to return the completed questionnaire after the program ended. The recruitment process continued for two months—February and March, 2019—and over 200 women attended each program; of which, 310 participants completed the questionnaire. Data from 281 participants were used for the statistical analysis after those in early pregnancy (*n *= 25) and those with incomplete data (*n* = 4) were excluded.

The survey questionnaire consisted of the PSQI and items pertaining to depressive symptoms and pregnancy stress. Demographic information regarding age, height, weight, education level, current employment status, household income, planned pregnancy, and expected due date was also collected. Current gestation was calculated based on the due date, and then divided into the mid- (< 28 weeks of gestation) and late (≥ 28 weeks of gestation) pregnancy groups.

The Korean version of the PSQI was used to assess participants’ sleep disturbances and quality [15]. This scale consists of seven components, and each of these components is scored between 0–3. Total scores range 0–21, with higher scores indicating poorer sleep quality. Since none of the participants reported using sleep medication, the component “Use of sleep medication” was removed during the analysis of the factor structure.

The Center for Epidemiologic Studies Depression scale was used to measure depressive symptoms in pregnant women [16, 17]. This 20-item scale measures depressive symptoms over the past week. It rates the symptom experience on a four-point Likert scale in terms of its frequency. Total scores range 0–60, with higher scores indicating more depressive symptoms. Cronbach’s alpha was 0.88 in this study.

The pregnancy stress scale was used to assess the perceived stress levels of the pregnant women in relation to pregnancy [18]. This scale was originally developed by Ahn [19] and was validated through a study involving pregnant Korean women [18]. The scale includes 20 items pertaining to six domains of stress related to the fetus, the expecting mother, her spouse, physical discomfort, household management, and parenting. Each item is rated on a five-point Likert scale. Total scores range 20–100 points, with higher scores indicating greater perceived stress. Cronbach’s alpha was 0.89 in this study.

### Statistical analysis

For the statistical analysis, IBM SPSS (v 26.0, IBM, Chicago IL, USA) was used. Descriptive statistics including frequency, mean, and standard deviation were computed to describe participants’ characteristics. Chi-square- and independent t-tests were used to determine the differences between the characteristics of women in mid- and late pregnancy and the component scores.

Exploratory factor analysis (EFA) was performed to investigate the factor structure using the principal component analysis with varimax rotation. This study’s sample size was considered appropriate for EFA [20]. Before conducting the analysis, the suitability for performing the factor analysis was assessed and confirmed. The study found that Bartlett’s test of sphericity, *p* < 0.001, and Kaiser–Meyer–Olkin’s measure of sampling adequacy was 0.67. To identify the number of meaningful factors, the scree plot and eigenvalues (> 1, Kaiser’s Rule) associated with each factor was considered. Sleep component with rotated factor loading ≥ 0.4 of absolute value was considered “dominant” and as the defining component for each specific factor. As an additional measure of convergent validity, the unadjusted correlation coefficients were computed between the PSQI scores, depressive symptoms, and pregnancy stress. The Cronbach’s alpha was used to test the internal consistency for the PSQI.

## Results

### Comparisons of participants’ characteristics and the PSQI components

Table 1 presents the characteristics of participants. The mean gestational age of mid- and late pregnancy was 22.6 weeks (SD 3.8) and 32.7 weeks (SD 3.1), respectively. The mean age of women in the mid- and late pregnancy group was 30.7 and 30.8 years, respectively. Most participants had a college degree or higher (86.2% for mid-pregnancy and 82.5% for later stages of pregnancy), and were currently unemployed (77.2% for mid-pregnancy and 71.6% for late pregnancy). Many participants reported a planned pregnancy (65.4% for mid-pregnancy and 66.2% for late pregnancy). Household income, current regular exercise, and pre-pregnancy Body Mass Index were similar between mid- and late pregnancy. Both depressive symptoms and pregnancy stress showed no significant difference between mid- and late pregnancy groups.

**Table 1:** Characteristics of participants

		Mid-pregnancy (*n* = 137)		Late pregnancy (*n *= 144)		
Variable (*n* = 281)		N (%) or Mean (SD)	Range	N (%) or Mean (SD)	Range	*p*
Age		30.7 (3.6)	20–38	30.8 (3.8)	21–48	.770
Gestational period (week)		22.6 (3.8)	14.3–27.9	32.7 (3.05)	28.0–39.0	< .001
Planned	No	47 (34.6)		48 (33.8)		.894
pregnancy	Yes	89 (65.4)		94 (66.2)		
Education	≤ High school	19 (13.9)		25 (17.5)		.538
	College	99 (72.3)		103 (72.0)		
	Graduate	19 (13.9)		15 (10.5)		.288
Employment	No	105 (77.2)		101 (71.6)		
	Yes	31 (22.8)		40 (28.4)		
Household income	Very low	15 (11.0)		19 (13.9)		.897
	Low	88 (64.7)		85 (62.0)		
	Middle	24 (17.6)		25 (18.2)		
	High	9 (6.6)		8 (5.8)		
Regular	No	83 (60.6)		76 (52.8)		.282
exercise	Yes	54 (39.4)		67 (46.5)		
Pre-pregnancy	Underweight	19 (13.9)		25 (17.4)		.142
BMI	Healthy weight	90 (65.7)		93 (64.6)		
	Overweight	9 (6.6)		16 (11.1)		
	Obese	19 (13.9)		10 (6.9)		
Sleep quality	Good (≤ 5)	59 (43.4)		46 (32.2)		0.53
	Poor (≥ 6)	77 (56.6)		97 (67.8)		
Depressive symptoms		9.5 (7.3)	0–34	10.9 (8.4)	0–38	.135
Pregnancy stress		51.7 (10.1)	28–82	51.3 (11.4)	20–95	.749

*SD* Standard deviation, *BMI* Body mass index

Table 2 shows the global and component scores of the PSQI in the mid- and late pregnancy groups. The mean global score significantly differed between mid- and late pregnancy groups with a score of 6.38 (SD 2.6) and 7.43 (SD 2.9), respectively. Except for sleep efficiency and daytime dysfunction, all other components were significantly different.

**Table 2:** Comparison of components between mid- and late pregnancy groups (*n *= 281)

Component	Mid-pregnancy		Late pregnancy		*P*
	Mean (SD)	Range	Mean (SD)	Range	
Subjective sleep quality	1.22 (0.62)	0–2	1.42 (0.62)	0–3	.008
Sleep latency	1.47 (0.92)	0–3	1.71 (1.00)	0–3	.035
Sleep duration	0.33 (0.70)	0–3	0.58 (0.91)	0–3	.010
Sleep efficiency	0.40 (0.83)	0–3	0.46 (0.89)	0–3	.533
Sleep disturbance	1.48 (0.57)	0–3	1.73 (0.58)	1–3	< .001
Daytime dysfunction	1.52 (0.88)	0–3	1.53 (0.75)	0–3	.895
Global score	6.38 (2.56)	1–14	7.43 (2.91)	2–15	.002

*SD* Standard deviation

### Factor structure of the PSQI

Table 3 Presents the exploratory factor analysis that revealed a two-factor solution in mid- and late pregnancy. For the mid-pregnancy group, the two-factor model explained 40.61% of the total variance. Factor 1, which included components of sleep latency, sleep duration, and sleep efficiency, was named “quantitative sleep quality,” whereas Factor 2, which included components of subjective sleep quality and sleep disturbance, was named “subjective sleep quality.” For the late pregnancy group, two factors were identified with a 43.66% total variance explained. Factor 1 included four components (sleep latency, duration, efficiency, and subjective sleep quality), and was labeled “perceived sleep quality.” Factor 2 included sleep disturbance and daytime dysfunction, and was labeled “daily disturbance.”

**Table 3:** Factor loading matrix for the PSQI between mid- and late pregnancy groups (*n* = 281)

	Mid-pregnancy		Late-pregnancy	
Component	Factor 1 Quantitative sleep quality	Factor 2 Subjective sleep quality	Factor 1 Perceived sleep quality	Factor 2 Daily disturbance
Subjective sleep quality	.253	**.750**	**.570**	.334
Sleep latency	**.445**	.327	**.546**	.225
Sleep duration	**.677**	.008	**.599**	-.025
Sleep efficiency	**.857**	-.093	**.824**	-.207
Sleep disturbance	-.045	**.420**	.384	**.449**
Daytime dysfunction	-.016	.354	-.097	**.628**
% of total variance	24.29	16.32	30.30	13.36

*PSQI* Pittsburgh Sleep Quality Index, Factor loadings ≥ 0.4 are shown in bold

### Convergent validity of the PSQI

Table 4 presents correlation coefficients between the global PSQI score, depressive symptoms, and pregnancy stress. The PSQI score was significantly positively correlated with the score of depressive symptoms and pregnancy stress (all p-value < 0.01).

**Table 4:** Correlation between the PSQI, depressive symptoms, and pregnancy stress of mid- and late pregnancy groups

Measures	Mid-pregnancy PSQI	Late pregnancy PSQI
Depressive symptoms	.574^**^	.475^**^
Pregnancy stress	.386^**^	.283^**^

*PSQI* Pittsburgh Sleep Quality Index

^*^p < .05

^**^p < .01

### Reliability of the PSQI

Table 5 shows the Cronbach’s alpha of the mid- and late pregnancy groups. Overall Cronbach’s alpha for the PSQI was 0.625, with 0.565 for the mid-pregnancy and 0.649 for the late pregnancy group.

**Table 5:** Cronbach’s alpha of mid-and late pregnancy groups (*n* = 281)

	Total	Mid-pregnancy	Late pregnancy
Component	Corrected item-total correlation^a^	Cronbach’s alpha if item is deleted^b^	Cronbach’s alpha if item is deleted^b^
Subjective sleep quality	.663^**^	0.477	0.561
Sleep latency	.702^**^	0.472	0.568
Sleep duration	.662^**^	0.477	0.580
Sleep efficiency	.642^**^	0.501	0.577
Sleep disturbance	.519^**^	0.569	0.597
Daytime dysfunction	.365^**^	0.600	0.717
Global score^c^		0.565	0.649

^a^The correlations between each component and the global PSQI score

^b^The PSQI’s Cronbach’s alpha reliability coefficient for internal consistency if the specific item is removed from the scale

^c^Overall Cronbach’s alpha (the overall reliability of the PSQI)

^**^p < .01

## Discussion

Sleep health of pregnant women, measured using a reliable and valid scale is important to provide a proper and timely intervention leading to optimal outcomes for expecting mothers and their children. This study assessed the quality of sleep among pregnant women in different pregnancy stages such as mid- and late pregnancy and compared the factor structure of the PSQI scale between two groups. The EFA revealed a two-factor structure model in both women in mid- and late pregnancy; however, each of the two factors in the mid- and late gestational period included different components of the PSQI, indicating different attributes of sleep quality concept.

Among women participated, the proportion of poor sleep quality (global score > 5) reported no statistical difference between the mid- (56.6%) and late (67.8%) gestational period; however, when the actual global score of the PSQI was compared, a significant difference was found. The average score of 7.43 in late pregnancy was significantly higher than the score of 6.38 in mid-pregnancy. This study also supports that the disturbance in sleep becomes worse with the progression of gestation [1, 3, 21, 22]. Using the same PSQI score, the average score was 4.5 in Peruvian pregnant women [13], 5.2 in US pregnant women [8], and 6.1 in a meta-study conducted with pregnant women [1]. Pregnant Korean women seem to report a somewhat higher score and lower sleep quality and this result needs to be investigated to understand the contributing factors leading to decreased sleep quality.

In this study, no participant reported using sleep medication, as generally, pregnant women seldom get prescriptions for it [23]. Thus, zero scores were given on this component and as such, the previously validated cut-off points of five needs to be carefully understood for the pregnant women. This is further supported by the finding that the use of sleep medication showed the lowest correlation with the global PSQI score of pregnant women [13]. For the pregnant women in the meta-analysis, the revised cut-off points were suggested to better differentiate good and poor sleepers [1].

The PSQI was originally developed as a single-factor scale and the construct of sleep quality was defined based on clinical judgment alone [11]. The PSQI was intended to measure the multifaceted nature of sleep quality, including quantitative and subjective aspects of sleep [12]. Zhong (2015) reported a three-factor model (sleep efficiency, sleep quality, medication) in Peruvian pregnant women [13] and a two-factor model (sleep quality and sleep disturbance) in US pregnant women who did not use sleep medication [1]. A two-factor structure has been the most common model of the scale in studies involving a variety of populations, such as healthy adolescents and middle-aged women experiencing hot flashes [12, 13, 24]. As such, in this study with mid- and late pregnancy women, the two-factor model was identified. However, importantly, the component in each factor in each gestational stage was not same.

For the mid-pregnancy group, three components of sleep duration, latency, and efficiency reflected Factor 1 (quantitative sleep quality) and two components, sleep quality and disturbance indicated Factor 2 (subjective sleep quality). However, for the late pregnancy group, the component of “subjective sleep quality” was additionally included in Factor 1 (perceived sleep quality) and daytime dysfunction was included in Factor 2 (daily disturbance). Given that the general characteristics of mid- and late pregnancy did not differ in this study, gestation can possibly be considered as one of the attributes contributing to factor structure change. These findings prove that the attributing component of sleep quality changes depending on the gestational stage in women. Interestingly, the component of daytime dysfunction was not attributed to the sleep quality of pregnant women in mid-pregnancy, indicating that daytime dysfunction did not contribute to the construct of sleep quality but was included in the later stage of pregnancy. This finding supports that the use of a single summed global score of all six components of the PSQI (sleep medication use excluded) might not efficiently capture the multi-dimensional nature of poor sleep quality. In this study, the verification of the factor structure is further examined, before using the PSQI scale.

This study demonstrated the convergent validity of the PSQI with evidence of significant positive correlations between the score of the PSQI and depressive symptoms. Pregnant women with poor sleep quality often reported experiencing more depressive symptoms [21, 25]. This study will aid medical professionals in correctly interpreting the scale's score and comprehending any factors influencing pregnant women's sleep quality. A more comprehensive and systematic screening of sleep health during pregnancy is thus required to avoid any health complications. The use of the PSQI scale is a reasonable screening approach that may provide clinicians with information on sleep disturbance. Thus, using it would help health care providers to offer individualized intervention to women with poor sleeping patterns and different gestational periods, thereby inducing better mental health and pregnancy outcomes [22].

The overall Cronbach's alpha of the PSQI was 0.63, higher than that of the study with Peruvian pregnant women [13]. Varying distributions of Cronbach's alpha values (0.57–0.83) have been reported using the PSQI scale [12, 13]. In general, Cronbach’s alpha is calculated based on the assumption that the factor loading values were the same, so each item had the same importance [26]. The multi-dimensionality of the PSQI seemed to cause a somewhat lower reliability in this study. The Cronbach’s alpha of this study showed similarity to prior studies that have reported multiple factor models [10, 13].

This study has several limitations. First, it employed a convenience sampling approach, and the sample may not have represented pregnant women in general. Owing to the cross-sectional nature of the study, changes in the factor structure of individual participants could not be warranted; thus, future studies should employ a longitudinal study design to obtain a more reliable result. EFA was used in this study to determine the structure of PSQI in pregnant Korean women. However, it was not possible to conduct confirmatory factor analysis (CFA) to verify whether the measurement tool was measuring by appropriately reflecting the dimension of the concept to be measured due to insufficient participants. Therefore, based on the structure discovered in this study, CFA is required in future studies with a sufficient number of pregnant women participants. Furthermore, in the future, factors affecting the sleep quality of women in mid-and late pregnancy need to be compared and subsequently, timely intervention for pregnant women needs to be developed.

## Conclusion

This is the first study that compared the factor structure of the PSQI in different gestational stages in pregnant Korean women. A two-factor model in mid- and late pregnancy was identified but the component included in each factor was different. For women in mid-pregnancy, daytime dysfunction was not attributed to sleep quality. The results imply that the attributing component of sleep quality changes according to gestational stage. The Korean version of the PSQI was found to have good validity in assessing sleep disturbances among pregnant women, with a positive correlation of the PSQI score with depressive symptoms. Proper understanding of sleep assessment scores in pregnant women during different gestational periods would help health care providers support pregnant women to ensure good quality sleep for them, thus securing their well-being and healthy mental status.

## Data Availability

The datasets used and/or analyzed during the current study are available from the corresponding author on reasonable request. Because data analysis is still being conducted.

## Abbreviations

BMI: Body mass index
CFA: Confirmatory factor analysis
EFA: Exploratory factor analysis
IRB: Institutional Review Board
PSQI: Pittsburgh Sleep Quality Index
SD: Standard deviation
